# Alveolar–Arterial Gradient Is an Early Marker to Predict Severe Pneumonia in COVID-19 Patients

**DOI:** 10.3390/idr14030050

**Published:** 2022-06-15

**Authors:** Giuseppe Pipitone, Marta Camici, Guido Granata, Adriana Sanfilippo, Francesco Di Lorenzo, Calogero Buscemi, Antonio Ficalora, Daria Spicola, Claudia Imburgia, Ilenia Alongi, Francesco Onorato, Caterina Sagnelli, Chiara Iaria

**Affiliations:** 1 Infectious Disease Unit, ARNAS Civico-Di Cristina, Piazza Leotta 5, 90100 Palermo, Italy; adriana.sanfilippo@arnascivico.it (A.S.); francesco.dilorenzo@arnascivico.it (F.D.L.); calogero.buscemi@arnascivico.it (C.B.); anfical@tin.it (A.F.); dariaspicola@yahoo.it (D.S.); claudiaimburgia@gmail.com (C.I.); ilenia.alongi@arnascivico.it (I.A.); francesco.onorato@arnascivico.it (F.O.); iaria.chiara@gmail.com (C.I.); 2 National Institute of Infectious Diseases Lazzaro Spallanzani, Via Portuense 292, 00149 Rome, Italy; marta.camici@inmi.it (M.C.); guido.granata@inmi.it (G.G.); 3 Infectious Disease Unit, University Hospital Luigi Vanvitelli, Vico Luigi De Crecchio, 80138 Naples, Italy; caterina.sagnelli@unicampania.it

**Keywords:** COVID-19, severity marker, alveolar–arteriolar oxygen gradient

## Abstract

Background: One of the main challenges in the management of COVID-19 patients is to early assess and stratify them according to their risk of developing severe pneumonia. The alveolar–arterial oxygen gradient (D(A-a)O_2_) is defined as the difference between the alveolar and arteriolar concentration of oxygen, an accurate index of the ventilatory function. The aim of this study is to evaluate D(A-a)O_2_ as a marker for predicting severe pneumonia in COVID-19 patients, in comparison to the PaO_2_/FiO_2_. Methods: This retrospective, multicentric cohort study included COVID-19 patients admitted to two Italian hospitals between April and July 2020. Clinical and laboratory data were retrospectively collected at the time of hospital admission and during hospitalization. The presence of severe COVID-19 pneumonia was evaluated, as defined by the Infectious Diseases Society of America (IDSA) criteria for community-acquired pneumonia (CAP). Patients were divided in severe and non-severe groups. Results: Overall, 53 COVID-19 patients were included in the study: male were 30/53 (57%), and 10/53 (19%) had severe pneumonia. Patients with severe pneumonia reported dyspnea more often than non-severe patients (90% vs. 39.5%; *p* = 0.031). A history of chronic obstructive pulmonary disease (COPD) was recalled by 5/10 (50%) patients with severe pneumonia, and only in 6/43 (1.4%) of non-severe cases (*p* = 0.023). A ROC curve, for D(A-a)O_2_ >60 mmHg in detecting severe pneumonia, showed an area under the curve (AUC) of 0.877 (95% CI: 0.675–1), while the AUC of PaO_2_/FiO_2_ < 263 mmHg resulted 0.802 (95% CI: 0.544–1). D(A-a)O_2_ in comparison to PaO_2_/FiO_2_ had a higher sensibility (77.8% vs. 66.7%), positive predictive value (75% vs. 71.4%), negative predictive value (94% vs. 91%), and similar specificity (94.4% vs. 95.5%). Conclusions: Our study suggests that the D(A-a)O_2_ is more appropriate than PaO_2_/FiO_2_ to identify COVID-19 patients at risk of developing severe pneumonia early.

## 1. Introduction

Since 31 December 2019, when the World Health Organization (WHO) was informed of an outbreak of respiratory disease affecting the city of Wuhan, the world has been shaken by the most profound health crisis in several decades [1]. Coronavirus Disease 2019 (COVID-19), caused by the Severe Acute Respiratory Syndrome Coronavirus 2 (SARS-CoV-2), has spread rapidly worldwide. COVID-19 patients often present a mild illness, but approximately 14% develop a severe disease which requires hospitalization and oxygen support. Moreover, 5% require admission to an intensive care unit [2]. The proper stratification of patients at hospital admission, according to their severity, is crucial for providing an early treatment, as well as for improving their management [3].

Therefore, one of the main challenges in the management of COVID-19 is to stratify patients early according to their risk of clinical deterioration. The alveolar–arteriolar oxygen gradient (D(A-a)O_2_) is defined as the difference between the alveolar and arteriolar concentration of oxygen, and it is a high accurate index of pulmonary function. Indeed, D(A-a)O_2_ describes all parameters involved in the phenomenon: (i) the amount of oxygen administered to the patient (FiO_2_), (ii) the atmospheric pressure (P_atm_), (iii) the partial oxygen pressure in arterial blood and the airway’s pressure of gaseous H_2_O (P_H2O_), (iv) the alveolar pressure of CO_2_ (PACO_2_), and (v) the respiratory quotient (R). 

It is described by the equation: D(A-a)O_2_ = [FiO_2_ × (P_atm_ − P_H2O_) − PACO_2_/R] − PaO_2_.

D(A-a)O_2_ is automatically calculated by a blood gas analyzer, and normally its value is between 5–10 mmHg.

It increases in the case of an alveolar–capillary membrane alteration (e.g., interstitial pneumonia) or ventilation/perfusion (V/Q) ratio impairment (e.g., pulmonary embolism, severe pneumonia etc.). As noted, all of the above are aspects of COVID-19 pneumonia [4].

D(A-a)O_2_ is automatically calculated in blood gas analysis (BGA), and has been proposed as an early marker of respiratory insufficiency.

The arterial oxygen partial pressure (PaO_2_) to fractional inspired oxygen (FiO_2_) ratio (PaO_2_/FiO_2_) is currently used to evaluate the severity of hypoxia in patients who require oxygen supplementation. A PaO_2_/FiO_2_ > 300 mmHg identifies a normal lung function [5]. The PaO_2_/FiO_2_ ratio was included in the definition of acute respiratory distress syndrome (ARDS) according to the Berlin criteria, and is globally used to stratify the severity of respiratory insufficiency [6].

However, PaO_2_/FiO_2_ ratio has some limitations. Firstly, PaO_2_/FiO_2_ depends on the clinicians FiO_2_ setting, so, when the oxygen flow is increased without a clear clinical need, the PaO_2_/FiO_2_ ratio can dramatically decrease, even if the pulmonary ventilation is little impaired. Secondly, PaO_2_/FiO_2_ does not consider the alveolar pCO_2_ value, that is, an indirect measure of the patient’s respiratory effort (e.g., tachypnea that can cause hypocapnia), reflecting the subjective severity of the respiratory insufficiency.

Finally, PaO_2_/FiO_2_ cannot provide information on pulmonary V/Q. Therefore, PaO_2_/FiO_2_ are less performant than D(A-a)O_2_ in discriminating between types of respiratory insufficiency (e.g., pump vs. pulmonary insufficiency). Moreover, COVID-19 patients who are not responding to a gradual incrementation of the oxygen flow can benefit from the early use of continuous positive airway pressure (CPAP) or non-invasive ventilation (NIV) [7,8,9,10,11].

As noted, COVID-19 pneumonia is characterized by both an alveolar and vascular damage [3]. Even though, few studies have been performed to assess the accuracy of D(A-a)O_2_ in early identifying severe COVID-19 pneumonia, and, as far as we know, no previous studies have compared the performance of D(A-a)O_2_ and PaO_2_/FiO_2_ in this context [12,13,14,15,16,17].

The aim of our study is to evaluate the diagnostic appropriateness of D(A-a)O_2_ to early predict respiratory deterioration in COVID-19. In addition, we compared the performance of D(A-a)O_2_ and PaO_2_/FiO_2_ in predicting pneumonia severity.

## 2. Materials and Methods

A retrospective, multicentric, case-control study was performed in two acute-care Italian hospitals: Infectious Disease Unit, ARNAS Civico Hospital in Palermo, and Infectious Disease Unit, Vanvitelli Hospital in Naples. All COVID-19 patients (≥18 years old) admitted to hospital between April and July 2020 were enrolled in the study. Patients affected by SARS-CoV-2 pneumonia, described as the presence of infiltrates on a computer tomography (CT) scan plus the positivity of the nasopharyngeal swab for the virus, were enrolled. Demographical, epidemiological, clinical, and laboratory data were retrospectively collected from the clinical records of each patient. Risk factors for severe COVID-19, as well as signs and symptoms at onset, were also collected. D(A-a)O_2_ and PaO_2_/FiO_2_ were calculated on the first blood gas analysis (BGA) performed in the emergency department. Patients were divided in two groups, considering the development of (1) severe pneumonia or (2) non-severe pneumonia, according the 2019 Infectious Diseases Society of America (IDSA) criteria for community-acquired pneumonia [18] (Table 1). Quantitative variables are shown as median and interquartile ranges (IQR) 25–75%. Qualitative variables are shown as number and percentage. Quantitative variables were tested for normal distribution with a Shapiro–Wilk test, and a U Mann–Whitney test was used for non-normally distributed variables (α = 0.05) to compare severe and non-severe patients.

For categorical variables, a two-sided Fisher exact test (α = 0.05) was performed to compare the two groups. Odds ratio (OR) were calculated with Spearman’s rank correlation coefficient. receiver operating characteristic (ROC) curves with a 95% confidence interval (CI) were created, and an area under the curve (AUC) was calculated in order to evaluate the optimal cut-off of D(A-a)O_2_ and PaO_2_/FiO_2_ at admission to predict the development of severe pneumonia during hospitalization, as well as to confront the accuracy of the two tests. Post-hoc analyses were conducted to evaluate the statistical power of the difference between both D(A-a)O_2_ and PaO_2_/FiO_2_ values of the two groups (severe and non-severe), by using mean and standard deviation of continuous variables for two independent sample, α = 0.05 (Rosner B. Fundamentals of Biostatistics. 7th ed. Boston, MA: Brooks/Cole; 2011).

## 3. Results

Overall, the study included 53 COVID-19 patients, whose demographic and clinical characteristics are shown in Table 2; laboratory data are showed in Table 3.

Among the patients, 10/53 (19%) developed severe pneumonia, and 43/53 (81%) non-severe pneumonia. One patient from each group had one episode of pulmonary thrombo-embolism during hospitalization.

Males were statistically prevalent among severe COVID-19 patients, compared to non-severe patients (90% vs. 49%, *p* = 0.031).

Comorbidities did not differ between the two study groups, with the exception of COPD, which was more prevalent in severe pneumonia patients than in non-severe patients (50% vs. 14%; *p* = 0.023), as well as malignancy (20% vs. 2.3%; *p* = 0.088). Table 2. The OR for severe pneumonia were calculated: for COPD, the OR was 6.167 (95% CI: 1.36–27.92, *p* = 0.011), and for male sex, the OR was 9.426 (95% CI: 0.940–50.644, *p* = 0.018).

At hospital admission in the emergency department, 42/53 (80%) patients presented fever, and 26/53 (50%) reported dyspnea. Dyspnea was diagnosed at admission in 9/10 (90%) severe and 17/43 (39.5%) non-severe patients (*p* = 0.005) Table 2.

A total of three patients died during the hospital stay (3/53, 5.6%), and all of deceased had severe COVID-19 pneumonia.

Median PaO_2_/FiO_2_ values at first BGA was lower in severe COVID-19 patients, as compared to non-severe patients, without reaching a statistical significance. For severe patients, the results were PaO_2_/FiO_2_ 246 mmHg (IQR 104.7–376.7), and for non-severe patients, the results were 390 mmHg (IQR 321.6–432.1), *p* = 0.157. Median values of D(A-a)O_2_ at first BGA were significantly higher for severe patients compared to non-severe patients: 97.9 mmHg (IQR 49.9–241.7) and 28.6 mmHg (IQR 12.3–40.2), respectively, *p* < 0.001.

D(A-a)O_2_ and PaO_2_/FiO_2_ have been compared through the ROC curve analysis for the prediction of severe pneumonia development.

**Figure 1:**
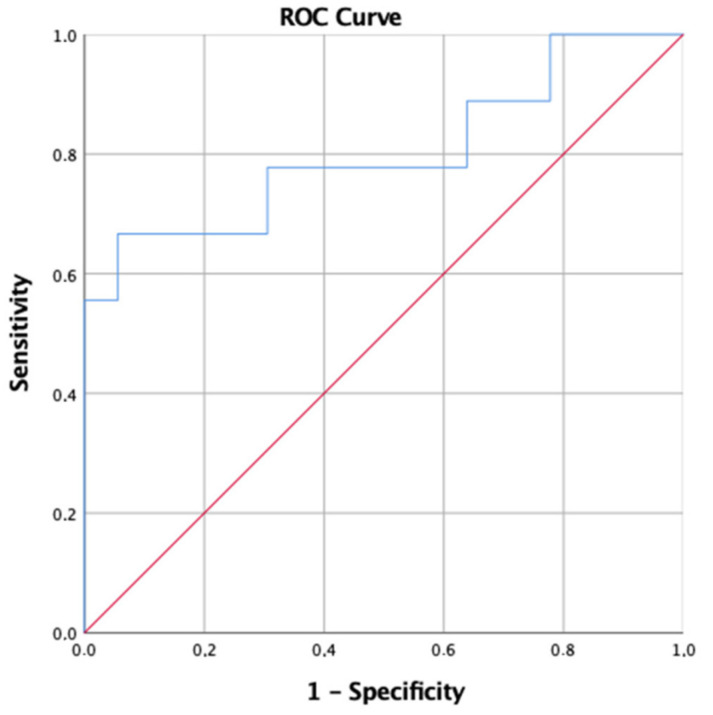
Receiver operating characteristic (ROC) curve comparing accuracy of PaO_2_/FiO_2_ at first BGA in relation to the prediction of severe pneumonia. Area under the curve (AUC): 0.802 (95% CI: 0.544–1).

For PaO_2_/FiO_2_, the more performant cut-off value for determining the occurrence of severe pneumoniae was <263 mmHg. Accordingly, the area under curve (AUC) resulted in a value 0.802, with a sensibility of 66.7% and a specificity of 94.5% (*p* = 0.001); positive predictive value (PPV) 71.4% and a negative predictive value (NPV) 91% (Figure 1).

**Figure 2:**
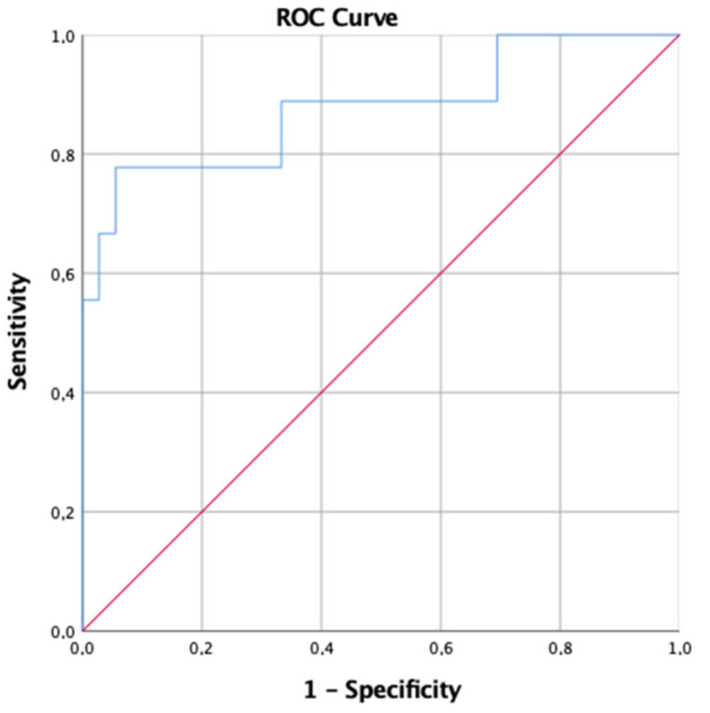
Receiver operating characteristic (ROC) curve comparing accuracy of D(A-a)O_2_ at first BGA in relation to the prediction of severe pneumonia. Area under the curve (AUC): 0.877 (95% CI: 0.675–1).

For D(A-a)O_2_, considering the cut-off value >60 mmHg for severe pneumonia, the AUC was 0.877, resulting in a sensibility of 77.8% and a specificity of 94.4%; PPV 75% and NPV 94% (Figure 2). Through a post-hoc analysis, the statistical power was calculated 87.7% for D(A-a)O_2_, and 88.2% for PaO_2_/FiO_2_. 

## 4. Discussion

During the COVID-19 pandemic, hospitals worldwide experienced overcrowding, leading to emergency and stressful situations [19]. A proportion of patients developed severe pneumonia, and one of the main challenges in their management was the early recognition of severe pneumonia itself, as well as the management of respiratory failure. 

A large cohort study which enrolled 10,131 elderly patients with COVID-19 showed that dyspnea was associated with a higher risk of hospitalization (aHR: 2.18; 95% CI: 2.02–2.36), mechanical ventilation (aHR: 2.95; 95% CI: 2.49–3.49), and mortality (aHR: 1.78; 95% CI: 1.53–2.07) [20]. The data confirm that dyspnea is associated with severe COVID-19 [20,21,22,23,24,25], as well as the association between COPD [6,26,27,28,29,30] and male sex [28,31] with severe SARS-CoV-2 infection.

Recently, a study by de Roos et al. supported the use of D(A-a)O_2_ in association with chest computed tomography scanning to identify patients in need of hospitalization at an early stage [4]. Moreover, D(A-a)O_2_ at first blood gas analyses at arrival in hospital was identified as a predictive marker of intensive care unit (ICU) admission [12,14], and of mortality in COVID-19 patients [19]. In a retrospective cross-sectional study among 213 patients admitted to ICU, D(A-a)O_2_ values were not sensitive or specific in predicting mortality [32].

Thus, as documented in the literature, in a non-ICU setting, D(A-a)O_2_ values could be used to identify patients in need of hospitalization [14], to identify patients at risk for ICU admission [12,14], and to predict mortality as part of a score [13]. However, if patients are already admitted to the ICU, perhaps D(A-a)O_2_ loses its predictive value [32], and this could be explained with the alteration of blood gases values among these patients.

The results of the present study align with the data of the consulted literature, and suggests that a low PaO_2_/FiO_2_ is a severity marker of SARS-CoV-2 infection [12,28], as well as D(A-a)O_2_.

As far as is known, this study is the first that directly compared the performance of the PaO_2_/FiO_2_ ratio and D(A-a)O_2_ in predicting severe pneumoniae (defined as IDSA guidelines on CAP) in non-ICU COVID-19 patients. The results indicate that D(A-a)O_2_ is more effective than the commonly used PaO_2_/FiO_2_ method to identify COVID-19 patients with a high risk of developing severe pneumonia.

According to the data, D(A-a)O_2_ is a better predictor for severe COVID-19 pneumonia than the PaO_2_/FiO_2_ at admission to hospital. Interestingly, we identify the best cut-off value for D(A-a)O_2_ as >60 mmHg for predicting severe pneumonia, with a sensibility of 77.8%.

The study has several limitations. Firstly, it is a retrospective study involving only two national hospitals, and it may not identify the complex heterogeneity of SARS-CoV-2 patients. Secondly, the sample size is relatively small, even if it has a good statistical power. Finally, we did not stratify for a variant of concern that presents a different pneumonia picture, virulence, or mortality.

## 5. Conclusions

D(A-a)O_2_ is an appropriate and useful marker to identify the risk of developing severe pneumonia in COVID-19 patients at an early stage. D(A-a)O_2_ had a higher predictive value in diagnosing severe COVID-19 compared to PaO_2_/FiO_2_, with a higher sensibility and a similar specificity. As a consequence, we support the routine use of D(A-a)O_2_ in COVID-19 emergency departments, considering patients with D(A-a)O_2_ >60 mmHg at admission as having a high risk of developing severe COVID-19 pneumonia. Furthermore, we support the use of D(A-a)O_2_ as a severity marker of pulmonary disease in non-ICU daily ward routines, as well as its application in clinical studies. Further research is needed, particularly in the setting of new SARS-CoV-2 variants of concern with different virulence and mortality rates.

## Figures and Tables

**Table 1:** IDSA criteria for severity in community-acquired pneumonia (CAP), based on 2019 guidelines.

IDSA Criteria for Severe CAP. One Major Criteria or Three or More Minor Criteria.
**Minor criteria**
Respiratory rate > 30 breaths/min PaO_2_/FIO_2_ ratio < 250
Multilobar infiltrates
Confusion or disorientation
Uremia (blood urea nitrogen level >20 mg/dL)
Leukopenia (white blood cell count, 4.000 cells/μL due to infection alone
Thrombocytopenia (platelet count, 100.000/μL)
Hypothermia (core temperature, <36 °C)
Hypotension requiring aggressive fluid resuscitation
**Major criteria**
Septic shock with need for vasopressors
Respiratory failure requiring mechanical ventilation

**Table 2:** Patients’ comorbidity, signs, and symptoms at presentation. Data are shown as median (interquartile range 25–75%), or number (percentage). Fisher exact tests were used (α < 0.05). COPD: chronic obstructive pulmonary disease; CKD: chronic kidney disease; Data are shown as a number (percentage), or median and interquartile range (IQR) 25–75%. PaO_2_/FiO_2_: arterial oxygen partial pressure (PaO_2_) to fractional inspired oxygen (FiO_2_) ratio. D(A-a)O_2_: alveolar–arteriolar oxygen gradient.

	Overall (*n* = 53)	Severe (*n* = 10)	Non Severe (*n* = 43)	*p*-Value
**Age in years (IQR 25–75)**	63 (49–75)	66.5 (62.8–73.8)	60 (47.5–74)	0.294
**Male Sex**	30 (56.6%)	9 (90%)	21 (49%)	0.031
**Caucasian**	51 (96.2%)	9 (90%)	42 (97.7%)	0.254
***Comorbidity***				
Hypertension	35 (66%)	7 (70%)	28 (65.1%)	0.719
Cardiovascular Disease	12 (22.6%)	4 (40%)	8 (18.6%)	0.677
COPD	11 (20.8%)	5 (50%)	6 (14%)	0.023
CKD	8 (15.1%)	3 (20%)	5 (14%)	0.163
Malignancy	3 (5.7%)	2 (20%)	1 (2.3%)	0.088
Diabetes Mellitus (type II)	6 (11.3%)	1 (10%)	5 (11.6%)	1
***Signs and Symptom***				
Fever	43 (82.1%)	8 (80%)	35 (81.4%)	1
Dyspnea	26 (49.1%)	9 (90%)	17 (39.5%)	0.005
Anosmia	7 (13.2%)	2 (20%)	5 (11.6%)	0.604
Dysgeusia	6 (11.3%)	2 (20%)	4 (9.3%)	0.315
Cough	26 (49.1%)	6 (60%)	20 (46.5%)	0.501
Diarrhea	4 (7.5%)	0 (0%)	4 (9.3%)	1
***Arterial Blood Gas analysis, median (IQR)***				
PaO_2_/FiO_2_ (mmHg)	379.5 (303.1–426.8)	246 (104.7–376.7)	390.5 (321.6–432.1)	0.157
D(A-a)O_2_ (mmHg)	33.6 (15.5–54.1)	97.9 (49.9–241.7)	28.6 (12.3–40.2)	<0.001
***Outcome***				
Death *n* (%)	3 (5.7%)	3 (30%)	0 (0%)	0.0051

**Table 3:** Laboratory data are showed as median (IQR 25–75), a Mann–Witney test was used (α < 0.05). WBC: white blood cells. CPR: C-reactive protein; AST: aspartate transaminases; ALT: alanine transaminases; LDH: lactate dehydrogenase; D(A-a)O_2_: alveolar–arteriolar gradient.

	Overall (*n* = 53)	Severe (*n* = 10)	Non-Severe (*n* = 43)	*p*-Value
**WBC (cell/** **μ** **L)**	6.7 (5.27–9.02)	6.9 (4.97–10.14)	6.5 (5.43–7.75)	0.869
**Neutrophils**	4.2 (3.09–6.06)	5.5 (2.48–9.26)	4.02 (3.3–5.12)	0.592
**Lymphocites**	1.25 (0.91–1.93)	0.76 (0.25–1.68)	1.27 (0.99–2.09)	0.432
**Platelets (cell/** **μ** **L)**	209 (165–251)	171 (109–250)	210 (184–251)	1
**D-Dimer (ng/mL)**	748.5 (402.2–1266)	499 (328–1200)	779 (442.5–1188)	0.689
**Creatinine (mg/dL)**	0.82 (0.74–0.95)	0.88 (0.75–1.46)	0.81 (0.68–0.9)	0.213
**CPR (mg/L)**	2.78 (0.95–8.12)	8.48 (0.9–12.8)	2.39 (0.59–5.43)	0.056
**LDH (UI/L)**	229 (183–325)	261 (235–527)	205 (167–321)	0.112
**AST (UI/L)**	29 (16–48)	42 (29–52)	28 (16–37)	0.071
**ALT (UI/L)**	28 (17–44)	40 (19–79)	25 (16–39)	0.334
**pO_2_**	80 (69.6–95.4)	69 (54.5–87)	82 (71–98.2)	0.204
**pCO_2_**	33 (31–35.65)	32.1 (31–43)	33 (30.85–35.23)	0.625

## Data Availability

Data available on request due to privacy restriction.

## References

[1] WHO, The World Bank Tracking Universal Health Coverage-2021 Global Monitoring Report, Geneva 2021. https://cdn.who.int/media/docs/default-source/world-health-data-platform/events/tracking-universal-health-coverage-2021-global-monitoring-report_uhc-day.pdf?sfvrsn=fd5c65c6_5&download=true.

[2] Yang X., Yu Y., Xu J., Shu H., Xia J., Liu H., Wu Y., Zhang L., Yu Z., Fang M. (2020). Clinical course and outcomes of critically ill patients with SARS-CoV-2 pneumonia in Wuhan, China: A single-centered, retrospective, observational study. Lancet Respir. Med..

[3] Prediletto I., D’Antoni L., Carbonara P., Daniele F., Dongilli R., Flore R., Pacilli A.M.G., Pisani L., Tomsa C., Vega M.L. (2021). Standardizing PaO_2_ for PaCO_2_ in P/F ratio predicts in-hospital mortality in acute respiratory failure due to COVID-19: A pilot prospective study. Eur. J. Intern. Med..

[4] Chu D.K., Kim L.H.-Y., Young P.J., Zamiri N., Almenawer S.A., Jaeschke R., Szczeklik W., Schünemann H.J., Neary J.D., Alhazzani W. (2018). Mortality and morbidity in acutely ill adults treated with liberal versus conservative oxygen therapy (IOTA): A systematic review and meta-analysis. Lancet.

[5] Ranieri V.M., Rubenfeld G.D., Thompson B.T., Ferguson N.D., Caldwell E., Fan E., Camporota L., Slutsky A.S., ARDS Definition of Task Force (2012). Acute Respiratory Distress Syndrome: The Berlin Definition. JAMA.

[6] Zhou F., Yu T., Du R., Fan G., Liu Y., Liu Z., Xiang J., Wang Y., Song B., Gu X. (2020). Clinical course and risk factors for mortality of adult inpatients with COVID-19 in Wuhan, China: A retrospective cohort study. Lancet.

[7] World Health Organization Living Guidance for Clinical Management of COVID-19. https://www.who.int/publications/i/item/WHO-2019-nCoV-clinical-2021-22021.

[8] National Institutes of Health COVID-19 Treatment Guidelines Panel. Coronavirus Disease 2019 (COVID-19) Treatment Guidelines. https://www.covid19treatmentguidelines.nih.gov/.

[9] Alhazzani W., Evans L., Alshamsi F., Møller M.H., Ostermann M., Prescott H.C., Arabi Y.M., Loeb M., Gong M.N., Fan E. (2021). Surviving Sepsis Campaign Guidelines on the Management of Adults with Coronavirus Disease 2019 (COVID-19) in the ICU: First Update. Crit. Care Med..

[10] Damiani E., Adrario E., Girardis M., Romano R., Pelaia P., Singer M., Donati A. (2014). Arterial hyperoxia and mortality in critically ill patients: A systematic review and meta-analysis. Crit. Care.

[11] Ni Y.-N., Wang Y.-M., Liang B.-M., Liang Z.-A. (2019). The effect of hyperoxia on mortality in critically ill patients: A systematic review and meta analysis. BMC Pulm. Med..

[12] Carlino M.V., Valenti N., Cesaro F., Costanzo A., Cristiano G., Guarino M., Sforza A. (2020). Predictors of Intensive Care Unit admission in patients with coronavirus disease 2019 (COVID-19). Monaldi Arch. Chest Dis..

[13] Kamran S.M., Mirza Z., Moeed H.A., Naseem A., Hussain M., Fazal I., Saeed F., Alamgir W., Saleem S., Riaz S. (2019). CALL Score and RAS Score as Predictive Models for Coronavirus Disease 2019. Cureus.

[14] de Roos M.P., Kilsdonk I.D., Hekking P.-P.W., Peringa J., Dijkstra N.G., Kunst P.W.A., Bresser P., Reesink H.J. (2021). Chest computed tomography and alveolar–arterial oxygen gradient as rapid tools to diagnose and triage mildly symptomatic COVID-19 pneumonia patients. ERJ Open Res..

[15] Guan W., Ni Z., Hu Y., Liang W., Ou C., He J., Liu L., Shan H., Lei C., Hui D.S.C. (2020). Clinical Characteristics of Coronavirus Disease 2019 in China. NEJM.

[16] Secco G., Salinaro F., Bellazzi C., La Salvia M., Delorenzo M., Zattera C., Barcella B., Resta F., Vezzoni G., Bonzano M. (2021). Can Alveolar-Arterial Difference and Lung Ultrasound Help the Clinical Decision Making in Patients with COVID-19?. Diagnostics.

[17] Farina G., Gianstefani A., Salvatore V., Anziati M., Baldassarri F., Beleffi M., Cannizzaro A.M., Casadei E., Fantini J., Tubertini E. (2020). Alveolar-to-arterial oxygen gradient: Role in the management of COVID-19 infection mild population. ResearchSquare.

[18] Metlay J.P., Waterer G.W., Long A.C., Anzueto A., Brozek J., Crothers K., Cooley L.A., Dean N.C., Fine M.J., Flanders S.A. (2019). Diagnosis and Treatment of Adults with Community-acquired Pneumonia. An Official Clinical Practice Guideline of the American Thoracic Society and Infectious Diseases Society of America. Am. J. Respir. Crit. Care Med..

[19] Prasad K., McLoughlin C., Stillman M., Poplau S., Goelz E., Taylor S., Nankivil N., Brown R., Linzer M., Cappelucci K. (2021). Prevalence and correlates of stress and burnout among U.S. healthcare workers during the COVID-19 pandemic: A national cross-sectional survey study. eClinicalMedicine.

[20] Huang C., Wang Y., Li X., Ren L., Zhao J., Hu Y., Zhang L., Fan G., Xu J., Gu X. (2020). Clinical features of patients infected with 2019 novel coronavirus in Wuhan, China. Lancet.

[21] Li K., Wu J., Wu F., Guo D., Chen L., Fang Z., Li C. (2020). The Clinical and Chest CT Features Associated with Severe and Critical COVID-19 Pneumonia. Investig. Radiol..

[22] Tian S., Hu N., Lou J., Chen K., Kang X., Xiang Z., Chen H., Wang D., Liu N., Liu D. (2020). Characteristics of COVID-19 infection in Beijing. J. Infect..

[23] Ruan Q., Yang K., Wang W., Jiang L., Song J. (2020). Clinical predictors of mortality due to COVID-19 based on an analysis of data of 150 patients from Wuhan, China. Intensive Care Med..

[24] Wu C., Chen X., Cai Y., Xia J., Zhou X., Xu S., Huang H., Zhang L., Zhou X., Du C. (2020). Risk Factors Associated with Acute Respiratory Distress Syndrome and Death in Patients with Coronavirus Disease 2019 Pneumonia in Wuhan, China. JAMA Intern. Med..

[25] Wang D., Hu B., Hu C., Zhu F., Liu X., Zhang J., Wang B., Xiang H., Cheng Z., Xiong Y. (2020). Clinical Characteristics of 138 Hospitalized Patients with 2019 Novel Coronavirus—Infected Pneumonia in Wuhan, China. JAMA.

[26] Ioannou G.N., Locke E., Green P., Berry K., O’Hare A.M., Shah J.A., Crothers K., Eastment M.C., Dominitz J.A., Fan V.S. (2020). Risk factors for hospitalization, mechanical ventilation, or death among 10 131 US Veterans with SARS-CoV-2 Infection. JAMA Netw. Open.

[27] Tang N., Li D., Wang X., Sun Z. (2020). Abnormal Coagulation parameters are associated with poor prognosis in patients with novel coronavirus pneumonia. J. Thromb. Haemost..

[28] Rod J.E., Oviedo-Trespalacios O., Cortes-Ramirez J. (2020). A brief-review of the risk factors for COVID-19 severity. Rev. Saúde Pública.

[29] Gao Y.-D., Ding M., Dong X., Zhang J.-J., Azkur A.K., Azkur D., Gan H., Sun Y.-L., Fu W., Li W. (2021). Risk factors for severe and critically ill COVID-19 patients: A review. Allergy.

[30] Takahashi T., Ellingson M.K., Wong P., Israelow B., Lucas C., Klein J., Silva J., Mao T., Oh J.E., Tokuyama M. (2020). Sex differences in immune responses that underlie COVID-19 disease outcomes. Nature.

[31] Camici M., Zuppi P., Lorenzini P., Scarnecchia L., Pinnetti C., Cicalini S., Nicastri E., Petrosillo N., Palmieri F., D’Offizi G. (2021). Role of testosterone in SARS-CoV-2 infection: A key pathogenic factor and a biomarker for severe pneumonia. Int. J. Infect. Dis..

[32] Singh A., Soni K.D., Singh Y., Aggarwal R., Venkateswaran V., Ashar M.S., Trikha A. (2022). Alveolar Arterial Gradient and Respiratory Index in Predicting the Outcome of COVID-19 Patients; A Retrospective Cross-Sectional Study. Arch. Acad. Emerg. Med..

